# Efficacy of postoperative analgesia with duloxetine in posthemorrhoidectomy pain: a prospective, randomized, double-blind and placebo-controlled trial

**DOI:** 10.1186/s12871-022-01908-x

**Published:** 2022-12-01

**Authors:** Marlus Tavares Gerber, Humberto Fenner Lyra, Thomas Rolf Erdmann, Fernanda Bomfati, Patrick Barcelos Gaspareto, Getúlio Rodrigues de Oliveira Filho

**Affiliations:** 1 University Hospital, Federal University of Santa Catarina, Maria Flora Pausewang St. S/N Campus Universitário – Trindade, Florianopolis, Santa Catarina 88036-800 Brazil; 2 Department of Surgery, Federal University of Santa Catarina, Florianopolis, Santa Catarina Brazil; 3 Erasto Gaertner Hospital, Curitiba, Parana Brazil

**Keywords:** Acute pain, Duloxetine, Hemorrhoidectomy, Morphine consumption, postoperative pain

## Abstract

**Background:**

To evaluate the effect of duloxetine when added to a multimodal analgesia regimen on posthemorrhoidectomy pain, opioid consumption, and side effects.

**Methods:**

Prospective, randomized, double-blind placebo-controlled trial. This study included 62 patients who underwent hemorrhoidectomy. The patients were randomly assigned to receive oral duloxetine 60 mg or placebo 2 h before and 24 h after surgery. The primary outcomes were pain intensity - measured on an 11-point visual analog pain scale - and cumulative morphine consumption at 12, 24, and 48 postoperative hours.

**Results:**

Fifty-two patients completed the study (25 in the duloxetine group and 27 in the placebo group). Pain scores did not differ between duloxetine and placebo: 4.5; 3.0 – 7.0 vs. 5.0; 3.5 – 7.0, *p* = 0.68 at 12 h, 3.0; 2.0 – 5.0 vs. 3.0; 2.0 – 5.0, *p* = 0.56 at 24 h, and 2.5; 1.75 – 3.75 vs. 1.5; 0.5 – 3, *p* = 0.08 at 48 h. Further, cumulative morphine consumption did not differ between the duloxetine and placebo groups: 4; 1.25 – 10.75 mg vs. 7; 1.0 – 12.0 mg, *p* = 0.68 at 12 h, 9.5; 2.0 – 17.5 mg vs. 8.0; 4.0 – 18.0 mg; *p* = 0.80 at 24 h, and 11.0; 2.0 – 27.0 mg vs. 10; 4.0 – 24.0 mg, *p* = 0.78 at 48 h. Side effects did not differ between the groups.

**Conclusions:**

Compared with placebo, duloxetine did not decrease pain intensity or morphine consumption during the first 48 h postoperatively.

**Trial registration:**

The study was retrospectively registered on the Brazilian Clinical Trials Registry (identifier: RBR-9pdgms, registration date: 08/10/2020).

## Background

Substantial progress in understanding acute pain mechanisms has been made in the last few decades. The neurotransmitters involved in primary afferent stimulation and the role of the spinal grey matter in amplifying and perpetuating acute pain are now better understood [1].

Consonant with the developing body of knowledge about pain mechanisms, multimodal analgesia is currently adopted for managing acute pain. Multimodal analgesia consists of using drug combinations to increase the analgesic effects and minimize the risks of adverse effects of the individual drugs. Currently, the most widely used multimodal analgesia approach for preventing posthemorrhoidectomy pain consists of the combined administration of opioids, non-steroidal anti-inflammatory drugs, local anesthetic infiltration, neuraxial anesthesia, and small doses of ketamine [2–4].

Duloxetine is a selective serotonin and norepinephrine reuptake inhibitor used to treat depression, anxiety disorders, diabetic neuropathy, and fibromyalgia. Both serotonin and norepinephrine play meaningful roles as neurotransmitters in the descending inhibitory pain pathways. Duloxetine has been found to inhibit central pain by potentiating serotonergic and noradrenergic inhibitory pathways [5, 6]. Several studies have demonstrated the efficacy of duloxetine to treat chronic pain [7, 8]. More recently, a few studies with conflicting results addressed the effects of short-term preoperative administration of duloxetine as part of multimodal analgesia regimens on postoperative (PO) pain in patients submitted to orthopedic, gynecological, or breast surgeries [9].

The effects of duloxetine added to a multimodal analgesia regimen to decrease posthemorrhoidectomy pain are unclear. We hypothesized that, in patients undergoing hemorrhoidectomy, oral administration of duloxetine at a dose of 60 mg, 2 h before and 24 h after surgery could decrease the intensity of pain in the first 48 h of the PO period, compared to placebo.

## Methods

### Trial design

This prospective, randomized, placebo-controlled, double-blind, parallel clinical trial was performed at the University Hospital of the Federal University of Santa Catarina, in Florianopolis, Brazil, from April 2019 through November 2020 after obtaining approval from the institutional review board (identifier: 2.722.942). Written informed consent was obtained from the participants. The study protocol was registered on the Brazilian Clinical Trials Registry (ReBEC platform, available at ensaiosclinicos.gov.br, identifier: RBR-9pdgms, registration date: 08/10/2020); the manuscript was written according to the 2010 Consolidated Standards of Reporting Trials (CONSORT) [10].

### Participants

The study included patients aged 18–65 years with symptomatic grades II through IV hemorrhoidal disease (HD), scheduled for elective hemorrhoidectomy under subarachnoid anesthesia. The exclusion criteria are listed in Table 1.

**Table 1:** Exclusion criteria

Patients younger than 18 years or older than 65 years	
Urgent or emergency surgery	
Pregnancy	
ASA class III, IV or V	
Past or present history of:	
Anal surgery	
Concomitant anorectal disease	
Coagulopathy	
Renal failure	
Hepatic failure	
Psychiatric illness	
Drug or alcohol abuse	
Chronic pain	
Opioid use	
Known allergy to duloxetine or any other drug used in the study	
Patients who refuse or have any contraindication to subarachnoid anesthesia	

*ASA* American Society of Anesthesiologists

Block randomization was performed before participant recruitment was conducted by an investigator not involved in any other study phase. Sixty cases were randomized in blocks of 6 (3:3) and allocated to the study groups (duloxetine or placebo) using random numbers generated using the www.randomizer.org website.

Identical capsules containing a placebo (500 mg of corn starch) or duloxetine (Velija™, Libbs Farmacêutica Ltda, São Paulo) 60 mg were prepared by the hospital pharmacy to ensure blinding of the participants, surgeon, anesthetist, and data collectors. Capsules were packed in opaque, sealed envelopes and sequentially numbered according to the randomization list provided by the hospital pharmacy. Envelopes were provided to the ward nurse after patient admission to the hospital and were opened at the study drug administration, assuring that neither the nurse administrating nor the patient receiving the capsule could presume the group assignment.

### Outcome measures

The primary study outcomes were postoperative pain intensity and patient-controlled analgesia (PCA) morphine consumption. Pain intensity was assessed on an 11-point visual analog scale (VAS), where zero meant no pain and ten meant the worst pain imaginable. Pain scores were assessed at the patient’s arrival to the post-anesthesia care unit (PACU) (time 0), and after 12, 24, and 48 PO hours. Additionally, patients were instructed to assess the pain at first PO defecation on the same VAS.

The cumulative morphine consumption was extracted from the PCA pump (Perfusor™ Space Infusion Syring Pump, B. Braun™, Melsungen) at the 12th, 24th, and 48th PO hours. Secondary outcomes were the frequency of adverse events attributable to duloxetine (nausea, vomiting, dry mouth, dizziness, urinary retention, headache, ileus, drowsiness, urticaria), recorded by ward nurses and investigators during the initial 48 PO hours, and the time to the first defecation.

### Perioperative management

Patients were admitted to the hospital the evening before surgery. Instructions on how to use the visual analog pain scale and the PCA pump were provided during the preoperative visit. No bowel preparation was performed. The study drug was administered orally 2 h before surgery and 24 h after the procedure by the nursing staff. Patients were premedicated with intravenous (i.v.) midazolam 0.03 mg.kg^− 1^ and fentanyl 1 μg.kg^− 1^ upon arrival to the operating room (OR), Cefoxitin 2 g was administered for perioperative antibiotic prophylaxis. Surgical anesthesia was provided with a spinal block with 10 mg of 0.5% hyperbaric bupivacaine. Ketoprofen 100 mg, dipyrone 2 g, and ondansetron 4 mg were administered at the end of surgery for preventive analgesia and postoperative nausea or vomiting prophylaxis.

The patients underwent closed hemorrhoidectomy in the jackknife position using a diathermal scalpel for resecting the three hemorrhoidal piles. After the procedure, the patients were transferred to the PACU.

Postoperative analgesia consisted of ketoprofen 100 mg at 12-hour intervals and dipyrone 2 g at six-hour intervals during the first 48 PO hours. Nausea and vomiting were treated with i.v. ondansetron 4 mg. Additionally, all patients used the i.v. PCA pump during the first 48 h after surgery using the following protocol: a) solution: morphine sulfate 1 mg.ml^− 1^, 50 mL; b) no background infusion; c) bolus demand: 1 mg; d) lock-out time: 5 min; e) maximum dose in 1 h: 8 mg.

Patients were discharged 48 h after surgery. Dipyrone 500 mg q.i.d. for 7 days, nimesulide 100 mg b.i.d. for 6 days, and tramadol 100 mg, for rescue analgesia were prescribed orally. A PO follow-up visit was scheduled for the eighth PO day. Patients were also instructed to return to the hospital in case of intractable pain, bleed, nausea or vomiting, urinary retention, or any other condition.

### Statistical analyses

The variables included in the analyses were age, sex, body mass index, ASA classification, tobacco use, HD degree, preoperative symptoms, duration of surgery, OR time, VAS pain scores, cumulative amount of morphine during the initial 48 PO hours, time to first defecation, and side effects. The distribution of continuous variables was assessed using the Kolmogorov-Smirnov, Shapiro-Wilk, and Lilliefors tests. Normally distributed continuous variables were compared between the groups using unpaired Student’s t-tests. The associations between categorical variables and treatments were assessed using the chi-square test (χ^2^) or Fisher exact tests. Mann-Whitney U tests were used to compare pain scores and cumulative morphine consumption between the groups at each measurement time point.

The effect sizes used for sample size estimations in this study were based on the findings of seven studies [5, 11–16] that had compared postoperative opioid consumption and pain at rest between patients receiving short-term perioperative duloxetine or placebo available at the time the study was conceived. Accordingly, an average 36% (SD = 13%) reduction in cumulative opioid consumption, measured as i.v. morphine equivalents, and a 14% (SD = 15%) between-group difference in VAS pain scores favoring duloxetine were reported at the 24th PO hour in these studies. Similar differences were assumed for the measurements of the 12th and 48th PO hours. These effect sizes were used to estimate sample sizes based on VAS pain score and cumulative morphine consumption, assuming the probabilities of type I error (alpha) = 5% and type II error (1 – beta) = 10%, and independent sample t-tests. A total of 52 patients allocated into two groups in a 1:1 ratio was estimated as the minimal sample size to detect the 14% between-group difference in VAS scores, while ten patients allocated into two groups in a 1:1 ratio were estimated as the minimal sample size to detect the 36% between group difference in the cumulative PCA morphine consumption. Considering possible losses, 60 patients were randomized.

Statistical analysis was performed using SPSS version 27.0 (SPSS Inc., Chicago, IL, USA). Sample size calculations were performed using the G*Power software [17]. Probabilities of type I error below 5% were considered significant. Continuous variables are represented as mean ± SD or as median and 25th – 75th percentiles. Categorical variables are summarized as frequencies and percentages.

## Results

**Fig. 1:**
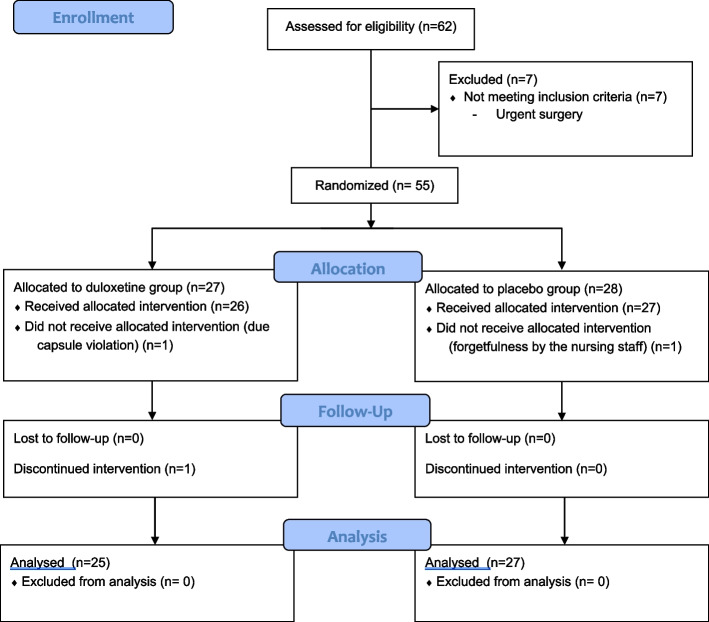
CONSORT Flow Diagram

Sixty-two patients were assessed for study eligibility between April 2019 and November 2020. Seven patients did not meet the criteria for inclusion in the study, and hence 55 patients were initially selected. Three patients were excluded during the study. Fifty-two patients, 27 in the placebo group and 25 in the duloxetine group, completed the study (Fig. 1).

The groups were homogeneous in terms of demographics and surgical characteristics. Also, no significant differences between groups regarding operative time or the time to the first defecation were observed (Table 2).

**Table 2:** Patients demographic features and symptoms of HD

	Duloxetine (***n*** = 25)	Placebo (***n*** = 27)	***p***-value
Age (years)	46.08 ± 11.07	50.41 ± 8.56	0.12
Sex (male/female)	14/11	15/12	0.97
BMI (kg.m^−2^)	27.53 ± 4.77	28.16 ± 5.26	0.65
Duration of the surgery (min)	40.0; 35.0 – 45.0	35.0; 32.5 – 40.0	0.06
Time in the OR (min)	60.0; 55.0 – 70.0	55.0; 50.0 – 60.0	0.17
ASA class			0.17
I	11 (44.0%)	7 (35.0%)	
II	14 (56.0%)	20 (65.0%)	
Smoking (past/present)	14 (56.0%)	12 (44.4%)	0.64
HD degree			0.65
II	4 (16.0%)	7 (25.9%)	
III	15 (60.0%)	15 (55.6%)	
IV	6 (24.0%)	5 (18.5%)	
Symptoms of HD			
Prolapse	24 (96.0%)	25 (92.6%)	1.00
Bleeding	23 (92.0%)	23 (85.2%)	0.67
Pain	18 (72.0%)	20 (74.1%)	1.00
Thrombosis	6 (24.0%)	10 (40.0%)	0.36
Itching	6 (24.0%)	6 (22.2%)	1.00
Difficulty with hygiene	6 (24.0%)	7 (25.9%)	1.00

Data are presented as mean ± SD, median; 25th - 75th percentiles or number (%)

*HD* hemorrhoidal disease, *BMI* body mass index, *min* minutes, *OR* operating room, *ASA* American Society of Anesthesiologists

VAS pain scores did not differ between duloxetine and placebo groups at 12, 24, and 48 postoperative-hour measurements (Table 3).

**Table 3:** Pain scores at rest in the first 48 hours

	Duloxetine (n = 25)	Placebo (***n*** = 27)	***p***-value
Time 0 - PACU	0.0; 0.0 – 0.0	0.0; 0.0 – 0.0	1.00
12 h PO	4.5; 3.0 – 7.0	5.0; 3.5 – 7.0	0.68
24 h PO	3.0; 2.0 – 5.0	3.0; 2.0 – 5.0	0.56
48 h PO	2.5; 1.75 – 3.75	1.5; 0.5 – 3.0	0.08

Data are presented as median; 25th - 75th percentiles

*PACU* post-anesthesia care unit, *h* hour, *PO* postoperative

Morphine consumption data of 17 patients (nine in the duloxetine group and eight in the placebo group) were lost due to technical reasons. Consequently, the analysis of morphine consumption was based on 16 patients from the duloxetine group and 19 patients from the placebo group, with no between-group difference (Table 4).

**Table 4:** Intravenous cumulative morphine consumption in milligrams

	Duloxetine (***n*** = 16)	Placebo (***n*** = 19)	***p***-value
Morphine consumption at 12 h PO	4.0; 1.25 – 10.75	7.0; 1.0 – 12.0	0.68
Morphine consumption at 24 h PO	9.5; 2.0 – 17.5	8.0; 4.0 – 18.0	0.80
Morphine consumption at 48 h PO	11.0; 2.0 – 27.0	10.0; 4.0 – 24.0	0.78

Data are presented as median; 25th - 75th percentiles

*h* hour, *PO* postoperative

The first postoperative defecation occurred after a median of 3 days in both groups (25th – 75th percentiles = 3 – 4 days, in duloxetine group, and 2 – 3 days in the placebo group). Median VAS pain scores at the first defecation were 10 (25th - 75th percentiles = 9 – 10) in the duloxetine group, and 10 (25th - 75th percentiles = 8 – 10) in the placebo group (*p* = 0.87). The prevalence of side effects attributable to duloxetine did not differ between groups (Table 5).

**Table 5:** Adverse effects attributable to duloxetine

	Duloxetine (***n*** = 25)	Placebo (***n*** = 27)	***p***-value
Nausea	11 (44.0%)	17 (62.9%)	0.17
Vomiting	6 (24.0%)	7 (25.9%)	0.87
Dry mouth	9 (36.0%)	4 (14.8%)	0.11
Dizziness	4 (16.0%)	3 (11.1%)	0.69
Urinary retention	2 (8.0%)	1 (3.7%)	0.60
Headache	0 (0.0%)	1 (3.7%)	1.00
Ileus	0 (0.0%)	1 (3.7%)	1.00
Drowsiness	1 (4.0%)	0 (0.0%)	0.48
Urticaria	0 (0.0%)	1 (3.7%)	1.00

Data are presented as frequency (%)

## Discussion

This study addressed the effect of duloxetine (60 mg) administered 2 h before and 24 h after surgery on posthemorrhoidectomy pain, morphine consumption, and side effects compared to placebo. The main findings were that duloxetine did not affect postoperative pain intensity or morphine consumption. Also, the incidence of side effects attributable to duloxetine was similar between the duloxetine and placebo groups.

Several studies that evaluated the postoperative analgesic effect of duloxetine have shown a significant effect [5, 12, 14, 16, 18–21], while other studies failed to detect any analgesic effect of duloxetine on postoperative pain [13, 15, 22]. Adding to the findings of the latter group of studies, a recent study from our group that assessed the effects of short-term preoperative duloxetine in patients submitted to colectomy also did not find any significant difference between placebo and duloxetine on postoperative PCA morphine consumption or visual analog pain scores [23].

Such contrasting results may result from methodological differences among the studies, including the type of anesthesia, type and extent of surgery, patient characteristics, surgical pain intensity, and the quality of background analgesia. Low-to-moderate VAS pain scores were found among patients in the placebo group of this study, further suggesting that adequate background analgesia provided to all patients may have blurred any existing analgesic effects of duloxetine. Although this may be an attractive explanation, in other studies in which low-to-moderate pain was reported in the placebo control groups, duloxetine has been associated with either significant or non-significant analgesic effects [9, 24]. The influence of baseline pain intensity on the analgesic effectiveness of duloxetine deserves further investigation.

Some methodological issues may limit the generalizability of our findings. First, results were based on postoperative opioid consumption and visual analog pain scores, imperfect surrogates for postoperative pain intensity. Both are affected by factors dependent on individual patients (e.g., culture, altruism, expectation, beliefs, education level, and age) [25]. Second, the same factors may affect the number of PCA analgesic requests and bias the total opioid consumption [26]. Third, although patients were educated preoperatively about the PCA pump and the visual analog pain scale, the pharmacologic effects of drugs administered in the postoperative period may have induced some information and response biases.

The current study should only be interpreted within the context of its limitations. This trial included patients with ASA physical status classes I and II aged 18 through 65 years submitted to closed hemorrhoidectomy; therefore, our findings should not be generalized to patients undergoing different surgical procedures or with other demographic features. Because the residual effect of bupivacaine spinal anesthesia may last up to 7 h in the sacral roots responsible for conducting anal pain [27], VAS scores assessed in PACU were zero in both groups. Other studies have detected an early (2 through 6 PO hours) analgesic effect of duloxetine in patients operated under general anesthesia [5, 14]. However, the cumulative PCA morphine consumption during the initial 12 PO hours did not differ between our groups, suggesting that pain intensity after the dissipation of bupivacaine analgesia was similar in both duloxetine and placebo groups. The loss of PCA morphine consumption data is an essential limitation of the study; nonetheless, the remaining available data far exceeded the a priori estimated sample size based on the morphine consumption outcome, so no case replacement was deemed necessary. Duloxetine may affect postoperative pain by its mood-modulating effect. Short-term preoperative administration of duloxetine has been associated with better quality of recovery after hysterectomy [11]. Such an outcome was not considered in this study.

## Conclusions

In conclusion, duloxetine in doses of 60 mg administered orally 2 h before and 24 h after surgery to patients undergoing hemorrhoidectomy does not decrease pain intensity or morphine consumption during the first 48 PO hours, compared to placebo. Similarly, pain at first defecation is not affected by duloxetine, and the prevalence of side effects was similar between the duloxetine and placebo groups. We highlight that this is a small pilot study using a heavy postoperative background analgesic regimen that may have obscured any analgesic effect of duloxetine. Another study from our group using the same background analgesic regimen did not provide evidence for any substantive analgesic effect of duloxetine [23]. Also, the available meta-analyses have found small effect sizes in postoperative pain scores or opioid consumption [9, 24], suggesting that further studies addressing the analgesic effect of duloxetine on postoperative pain in adult patients are no longer needed. Instead, perhaps new studies might target patients with conditions that demand either avoiding nonsteroidal anti-inflammatory drugs (e.g., renal failure, geriatric patients) or minimizing the dose of postoperative opioids (e.g., chronic obstructive pulmonary disease, sleep apnea patients).

## Data Availability

The data supporting the findings of this study are available from the corresponding author upon request.

## Abbreviations

PO: Postoperative
HD: Hemorrhoidal disease
ASA: American Society of Anesthesiologists
PCA: Patient-controlled analgesia
VAS: Visual analog scale
PACU: Post-anesthesia care unit
i.v.: Intravenous
OR: Operating room
b.i.d: Bis in die
SD: Standard deviation
BMI: Body mass index
